# Pregnancy Testing and Gynaecological History in Female Admissions to an Acute Psychiatric Unit

**DOI:** 10.1192/bjo.2026.11816

**Published:** 2026-06-30

**Authors:** Faadumo Osman, Imaan Islam, Rebecca Hall, Hayley Andrews

**Affiliations:** 1 Leicestershire Partnership Trust, Leicester, United Kingdom; 2 Leicester Medical School, Leicester, United Kingdom

## Abstract

**Aims:**

In a New Zealand psychiatric unit, of the 96 female patients (of reproductive age) started on sodium valproate, only 29 had pregnancy tests (1). Given the potential teratogenic effects associated with psychotropic medications alongside the potential for patients to have engaged in greater risk-taking behaviour, for instance when manic, it is essential that pregnancy and contraceptive status are considered in this patient group.

The purpose of this audit was to evaluate the screening for pregnancy and relevant gynaecological history of new female admissions (of reproductive age) to the Bradgate mental health unit in Leicester.

**Methods:**

A prospective audit was conducted of new female admissions of reproductive age to the Bradgate Mental Health Unit over a month. The patients’ medical records were evaluated and the data required for the audit was extracted from the records covering the first 2 weeks of admission.

A data collection tool was developed to record the information. This included whether a pregnancy test was conducted and whether a gynaecological history was taken.

**Results:**

A total of 28 new female admissions of reproductive age were admitted between 1st September and 1st October 2025. Of the 28 patients, only 14.3% (4/28) of patients had a pregnancy test within the first 2 weeks of admission. Additionally, 7.14% (1/28) of patients were asked if there is a chance of pregnancy during the clerking process and this patient subsequently had a pregnancy test. Only one patient was asked about their last menstrual period. None of the patients were asked about if they were on contraceptives or if they were sexually active. Finally, 53.57% (15/28) of patients were up to date with their cervical screening.

**Conclusion:**

In conclusion, this audit demonstrates that the assessment of pregnancy and related screening from women of a reproductive age was inconsistent and often missed entirely. Very few patients had documented pregnancy testing within 2 weeks of admission with none having sexual activity, contraceptive use or the last menstrual period dates recorded. This gap in assessment and documentation poses a risk to patient safety and suggests a need for a systems-based approach to standardise pregnancy screening. Potential strategies include raising staff awareness in the form of informative posters, presentations and potentially changes to prescribing software so that pregnancy status is considered prior to prescribing medications. Re-auditing following implementation of these interventions will determine if compliance has improved.

